# Factors which influence the cardiac surgeon's decision not to operate on patients referred for consideration of surgery

**DOI:** 10.1186/1749-8090-3-9

**Published:** 2008-02-26

**Authors:** Paul D Waterworth, Sing Y Soon, Rohith Govindraj, Rajesh Sivaprakasam, Mark Jackson, Antony D Grayson

**Affiliations:** 1 Department of Cardiothoracic Surgery, Wythenshawe Hospital, University of South Manchester NHS Foundation Trust, Manchester, UK; 2 Clinical Governance Department, The Cardiothoracic Centre, Liverpool, UK

## Abstract

**Background:**

The aim of this study was to document what proportion of patients referred for consideration of cardiac surgery are turned down, the reasons given for not operating and also to evaluate what happens to those patients who do not undergo surgery.

**Methods:**

382 elective patients referred for consideration of cardiac surgery to one of six consultant cardiac surgeons at Wythenshawe Hospital during a one year period from were included in the study. Data for those patients who underwent an operation were collected prospectively in a cardiac surgery database. The case notes of those patients who did not undergo an operation were reviewed to establish reasons given by surgeons for not operating. Patients were followed up to determine vital status at the end of the study period.

**Results:**

333 (87.2%) patients underwent an operation and 49 (12.8%) did not. 68% of patients turned down were thought to be too high-risk. 14% of patients did not fulfill symptomatic or prognostic criteria for surgery and in 8% of patients coronary artery surgery was thought ineffective due to poor distal vessels. 6% of patients declined an operation and 4% were thought to be more suitable for coronary angioplasty. Patients turned down for surgery had more renal dysfunction (p = 0.017), respiratory disease (p < 0.001) and peripheral vascular disease (p < 0.001), were more likely to have undergone prior heart surgery (p < 0.001) and to have poor left ventricular function (p = 0.003). Patients turned down for surgery had significantly higher EuroSCORE values compared to patients who underwent an operation: 5 versus 4 (p = 0.006). Freedom from death in the patients turned down for surgery at 1-, 6-, 12- and 24-months was 95.9%, 91.8%, 83.7% and 71.4% respectively, compared with 97.9%, 96.7%, 96.4% and 94.5% for the patients who underwent an operation (p < 0.001 [log-rank]). 14 of the 15 deaths that occurred in the turned down group occurred in the category considered too high-risk for surgery.

**Conclusion:**

12.8% of patients referred for consideration of cardiac surgery did not undergo an operation. Two thirds of patients not accepted for surgery were thought too high risk. Those patients who did not undergo an operation had a significantly worse mortality.

## Background

Since the Bristol Inquiry into paediatric cardiac surgical deaths in 1995 [1] there is greater awareness of the importance of clinical governance within hospitals in the UK. Emphasis has been placed on the importance of individual surgeons operative mortality results, particularly in cardiac surgery.

Political pressure resulted in the Society of Cardiothoracic Surgeons of Great Britain and Northern Ireland publishing operative mortality results for first-time coronary artery bypass surgery, for all consultant cardiac surgeons in the UK. These results were published in the *Fifth National Adult Cardiac Surgical Database Report 2003 *compiled by Keogh and Kinsman [2]. In this report it was thought inappropriate to give the exact mortality for each surgeon. Instead, surgeons were listed with a comment indicating whether they met the Society of Cardiothoracic Surgeons standards, which were defined as being acceptable if the surgeon fell within the 99.99% confidence intervals of the national average.

The Guardian Newspaper [3] went one step further and published surgeon-specific mortality data. Under the *Freedom of Information Act*, the Guardian wrote to the Chief Executives of all NHS Trusts with cardiac surgical units, insisting that surgeon specific mortality data for first-time coronary artery bypass surgery was submitted in whatever format it was available. The Guardian then went on to publish a mixture of crude data, observed mortality compared with expected mortality using the Parsonnet score [4], observed mortality compared with expected mortality using the additive EuroSCORE [5], observed mortality compared with expected mortality using the logistic EuroSCORE [6] and risk stratified mortality data. Although ground breaking, as this was the first publication in the UK of its kind which included named surgeons mortality results, allowing so many different formats led to an article which was confusing and thus allowed meaningless comparisons to be drawn between surgeons.

The surgeon-specific mortality data for first time coronary artery bypass surgery and first time aortic valve replacement for all consultant cardiac surgeons in the North West of England was published in the British Medical Journal [7]. In this paper risk stratified data was used.

In the current climate we feel an increasing proportion of those patients deemed high risk are likely to be denied surgery. This has already been seen in New York, USA [8].

With this in mind we set out to document what proportion of patients referred for consideration for cardiac surgery are turned down, the reasons given by surgeons for not operating on such patients, and also to evaluate what happens to those patients who do not undergo surgery.

## Methods

During a one year period from 1^st ^July 2002 to 30^th ^June 2003 all elective patients referred for consideration of cardiac surgery to one of six named consultant cardiac surgeons at Wythenshawe Hospital were recorded retrospectively. Patients were identified by the cardiac surgery outpatient computerised database as new patients. Only those patients referred via the Wythenshawe Hospital outpatient department were included in the study. Urgent and emergency inpatient referrals, and elective referrals from peripheral outpatient departments outside Wythenshawe Hospital, were excluded. This group of patients was then cross-referenced against a computerised list of patients who had undergone a cardiac surgical procedure form 1^st ^July 2002 to 31^st ^December 2004. In this way we were able to identify those patients who had undergone a cardiac surgical procedure and those who had not.

Each patient in either the operated or turned down groups had a data set collected, which included patient related, cardiac related and operation related factors allowing the calculation of an additive EuroSCORE, which equates to a predicted (or expected) mortality for each patient. The data for those patients who underwent an operation were collected prospectively into a cardiac surgery database during the patients admission. The case notes of those patients who did not receive an operation were retrospectively reviewed.

The reasons specified by the surgeon for not operating were documented from the outpatient records and were categorized into the following five groups:

1) Patient considered high risk.

2) Patients did not fulfill symptomatic or prognostic criteria for surgery. e.g. paucity of symptoms in combination with one or two vessel coronary artery disease or asymptomatic aortic stenosis with low gradient across valve.

3) Surgery was thought to be ineffective due to poor distal vessels

4) Angioplasty thought to be more appropriate.

5) Patient declined surgery.

Both the patients who underwent an operation and those who did not were followed up to determine whether they were alive at the end of the study period.

Ethical approval was obtained from The University of South Manchester NHS Foundation Trust Ethics Committee.

Continuous data are shown as median values with 25^th ^and 75^th ^percentiles, while categorical data are shown as a percentage. Data were compared with Wilcoxon rank sum tests and Chi-square tests as appropriate. Deaths occurring over time were described using Kaplan-Meier curves. All analysis was undertaken using SAS for Windows version 8.2 (SAS, Cary, NC).

## Results

**Figure 1:**
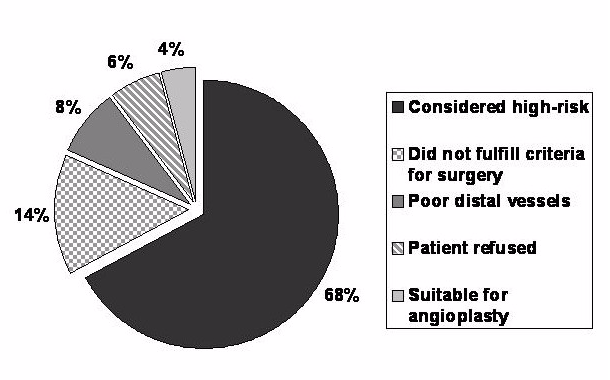
Reasons for surgical turndown from 49 patients.

During the study time period, 382 patients were referred for cardiac surgery. Of these, 333 (87.2%) patients underwent an operation, while the remaining 49 (12.8%) patients did not. The number of patients and rate of turndown by procedure are shown in Table 1. Figure 1 shows the reasons why patients were not accepted for surgery.

**Table 1:** Number of patients and rate of turndown by procedure

	Number of patients referred	Number of patients turned down	Turndown Rate
Isolated CABG	238	32	13.5%
Isolated Valve	82	12	14.6%
CABG+Valve	34	3	8.8%
Other	28	2	7.1%
Total	382	49	12.8%

Table 2 shows the patient characteristics depending on whether the patient underwent an operation or not. Patients turned down for surgery had more co-morbidity (renal dysfunction, respiratory disease, peripheral vascular disease), were more likely to have undergone prior heart surgery and have poor left ventricular function. Patients turned down for surgery were also on average 3 years older, although this just failed to reach statistical significance (p = 0.076).

**Table 2:** Patient characteristics depending on whether the patient underwent an operation or was turned down

	Turndown (n = 49)	Operated (n = 333)	p-Value
Age (years)	69 (61 – 74)	66 (58 – 72)	0.076
Female (%)	24.5	24.9	0.95
Diabetes (%)	12.2	12.9	0.89
Renal dysfunction (%)	6.1	1.2	0.017
Respiratory disease (%)	34.7	12.0	<0.001
Peripheral vascular disease (%)	24.5	4.8	<0.001
Cerebrovascular disease (%)	8.2	14.4	0.23
Endocarditis (%)	0	0.9	0.51
Recent MI (%)	0	1.8	0.34
EF 30–50% (%)	24.5	21.6	0.65
EF <30% (%)	12.2	3.0	0.003
Prior heart surgery (%)	14.3	2.7	<0.001

**Figure 2:**
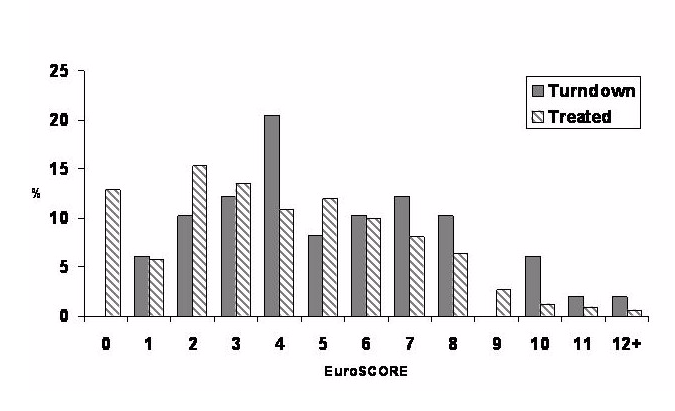
Distribution of additive EuroSCORE values depending on whether the patient underwent an operation or was turned down.

The difference in risk profile, as measured by the additive EuroSCORE, is shown in Figure 2. Patients turned down for surgery had significantly higher EuroSCORE values compared to patients who underwent an operation (5 [25^th ^and 75^th ^percentiles: 3–7] versus 4 [25^th ^and 75^th ^percentiles: 2–6]; p = 0.006). The proportion of patients turned down with a EuroSCORE > 5 was 42.9% compared with 29.7% in the operated group (p = 0.064).

**Figure 3:**
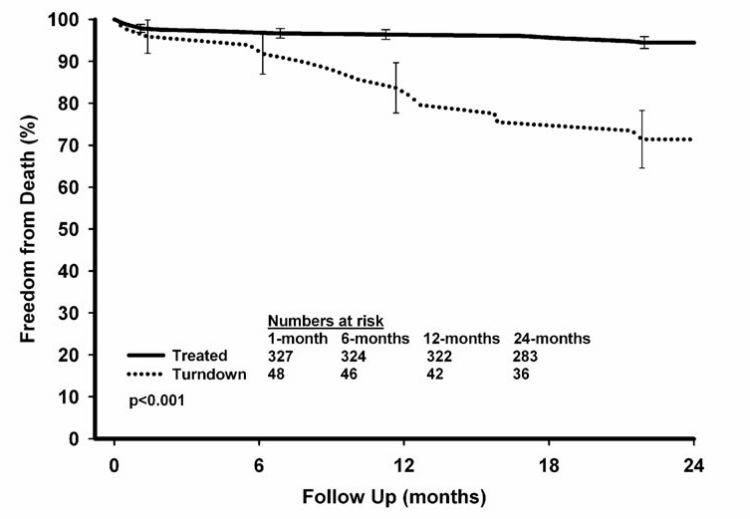
Observed survival.

**Figure 4:**
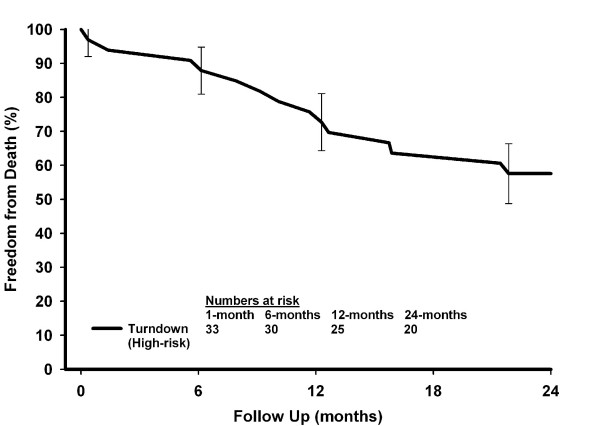
Observed survival in high-risk turndown patients.

Thirty-two (8.4%) deaths occurred during the study with a total follow-up period of 8,495 patient-months (mean follow-up of 22-months). Freedom from death in the patients turned down for surgery at 1-, 6-, 12- and 24-months was 95.9%, 91.8%, 83.7% and 71.4% respectively, compared with 97.9%, 96.7%, 96.4% and 94.5% for the patients who underwent an operation (Figure 3; p < 0.001 [log-rank]). On further analysis of the deaths that occurred in the turndown group, we found that 14 of the 15 deaths had occurred in the group marked as too high-risk for surgery. The remaining death occurred in a patient who declined surgery. Freedom from death in the turndown high-risk group at 6-, 12-, and 24-months was 87.9%, 72.7%, and 57.6% respectively (Figure 4).

## Discussion

12.8% of patients referred to a single cardiac surgical unit for consideration of cardiac surgery did not undergo an operation. Two thirds of those patients not accepted for surgery were thought to be too high risk for an operation. Those patients not accepted for surgery had more co-morbidity (i.e. renal dysfunction, respiratory disease and peripheral vascular disease) and were more likely to have undergone prior heart surgery and have poor left ventricular function. Those patients who did not undergo an operation had a significantly worse freedom from death at 1-, 6-, 12-, and 24-months after the decision not to operate, compared with those operated upon. Of course we do not know what the outcome would have been had these patients undergone surgery.

It can be seen in Figure 2 that a significant proportion of patients with Euroscores of 1–4 were turned down, in addition to those patients with a Euroscore of 5 or greater. Hence, it would appear that the Euroscore is inadequate when assessing individual patient risk, and that the surgeon relies on his personal assessment of each patient.

It is clear from these results that surgical risk is a significant factor taken into consideration in patients who are not accepted for a cardiac surgical procedure. This study has however documented one 'snap shot' in time, at a single institution, and it is difficult to know whether the number of patients not accepted for an operation would have been fewer prior to 1995 when cardiac surgery started to be placed under closer scrutiny.

To our knowledge this is the first study to attempt to quantify the number of patients referred for cardiac surgery who are considered unsuitable, to analyse the reasons given for not operating and to investigate the outcome of these patients. At the inception of this study we hoped to compare this study period with an earlier period prior to the widespread anticipation of the publication of surgeon specific mortality data. Due to the retrospective nature of the data collected this was not possible as there was no computerized data base for the earlier period.

This study has demonstrated that a significant number of patients are not accepted for cardiac surgery because of surgeons' concern about surgical risk. It has also shown that many of those patients who are referred for consideration of cardiac surgery, and do not receive an operation, ultimately have a poor outcome.

We believe that the ongoing publication of surgeon-specific data is likely to result in fewer "high risk" patients being offered an operation. Paradoxically it is usually these patients who have most to gain from an operation in terms of increased life expectancy and an improvement in quality of life.

One possible solution to this problem would be the publication of unit-specific mortality rather than surgeon-specific mortality. This is likely to offer patients the reassurance about quality of care and surgery at any particular centre, and still allow surgeons to offer high-risk patients the opportunity of a better future.

It is accepted that there is a wide variation in surgical practice: what one surgeon turns down, another may accept for surgery. We would therefore suggest that, in order to minimise the number of high-risk patients who are not accepted for surgery, any patient who is turned down for cardiac surgery is discussed with at least one other colleague so that a collective decision can be made. This already happens, to some extent, but the process could be improved and formalised.

It would be useful for all cardiac surgical units in the UK to prospectively collect data on all patients who are referred for consideration of cardiac surgery. Only by collecting this data on these patients, as well as those patients who receive an operation, will we truly know how well one surgeon, or one unit, compares with another.

Further analysis of the outcome of subgroups (e.g. coronary artery bypass surgery and aortic valve replacement) of high-risk patients who are not accepted for surgery is indicated.

## Competing interests

The author(s) declare that they have no competing interests.
